# Pharmacogenomics predictors of aromatic antiepileptic drugs-induced SCARs in the Iraqi patients

**DOI:** 10.1016/j.heliyon.2024.e41108

**Published:** 2024-12-18

**Authors:** Ali Fadhel Ahmed, Dzul Azri Mohamed Noor, Majeed Arsheed Sabbah, Nur Fadhlina Musa, Nur Aizati Athirah Daud

**Affiliations:** a Discipline of Clinical Pharmacy, School of Pharmaceutical Sciences, Universiti Sains Malaysia, 11800, USM Pulau Pinang, Malaysia; b Human Genome Centre, School of Medical Sciences, Universiti Sains Malaysia Health Campus, 16150, Kubang Kerian, Kelantan, Malaysia; c Forensic DNA for Research and Training Centre, Alnahrain University, Baghdad, 64074, Iraq

**Keywords:** Severe cutaneous adverse reactions, SJS, *HLA*, Pharmacogenetics, AED

## Abstract

**Introduction:**

Severe cutaneous adverse reactions (SCARs) are life-threatening and often linked to antiepileptic drugs (AEDs). Common types of SCARs include Stevens-Johnson syndrome (SJS), toxic epidermal necrolysis (TEN), and drug reaction with eosinophilia and systemic symptoms (DRESS). Immune-mediated mechanisms involving human leukocyte antigen (*HLA*) alleles have been implicated in the pathogenesis of this reaction. This study examines the association between specific *HLA* alleles (*HLA-A, -B*, and -*DRB1*) and AED-induced SCARs in the Iraqi population.

**Methodology:**

A total of 50 patients diagnosed with SCARs and 90 tolerant controls were recruited from Dr. Saad Al-Wattari Hospital for Neurological Sciences and Baghdad Hospital - Medical City. *HLA* genotyping was performed using PCR-SSO method from peripheral blood samples. Statistical comparisons were made using the *t*-test or chi-square test, while univariate logistic regression with Bonferroni's correction (p < 0.05) were used to assess associations between *HLA* alleles and SCARs.

**Results:**

Among the patients, SJS was the most prevalent type of SCARs observed. Analysis of *HLA* allele frequencies revealed significant associations between specific alleles. *HLA-A∗02:01* was found to be significantly associated with a lower risk of AED-induced SJS (OR = 0.36; 95 % CI: 0.13–0.97), while *HLA-A∗24:02* and *HLA-B∗15:02* were associated with an increased risk of AED-induced SJS (OR = 3.60; 95 % CI: 1.21–10.72 and OR = 4.41; 95 % CI: 1.18–16.47, respectively). For AED-induced TEN, *HLA-A∗01:02*, *HLA-B∗15:02,* and *HLA-B∗52:01* showed significant associations (OR = 6.92; 95 % CI: 1.39–34.37 and OR = 6.55; 95 % CI: 1.62–26.52, respectively), with *HLA-DRB1∗03:01* being highly significant (OR = 5.09; 95 % CI: 1.72–15.00). Additionally, *HLA-B∗40:02* was strongly associated with AED-induced DRESS (OR = 29.33; 95 % CI: 3.50–245.32).

**Conclusion:**

This study identifies key *HLA* alleles associated with AED-induced SCARs in the Iraqi population. These findings could facilitate personalized medicine approaches, aiding in better prediction and prevention of SCARs in AED therapy.

## Highlights

• Markers in HLA-A, HLA-B, and HLA-DRB1 genes are linked to severe cutaneous adverse reactions to antiepileptic drugs in Iraqis.
• Genetic screening for specific HLA markers can help prevent adverse reactions to antiepileptics, enhancing patient safety.
• Pharmacogenetic screening prevents drug adverse reactions, advancing personalized epilepsy treatment in diverse populations.

## 1 Introduction

Drug reactions typically occur due to immune-mediated mechanisms. In the United States of America, there is evidence linking certain drugs to over 100,000 deaths each year, making drug reactions the sixth leading cause of death. Similar statistics are observed in the United Kingdom [1,2].

Several antiepileptic drugs (AEDs) are commonly associated with adverse skin reactions [3], [4,5]. Severe and life-threatening skin reactions are referred to as severe cutaneous adverse reactions (SCARs) [6]. SCARs which occur following the use of certain drugs are a type of delayed hypersensitivity reaction that is idiosyncratic, unpredictable and dose-independent. SCARs account for 15–20 % of all adverse drug reactions, which include Stevens-Johnson syndrome (SJS), toxic epidermal necrolysis (TEN) and drug reaction with eosinophilia and systemic symptoms (DRESS).

Statistics indicate that the annual incidence of SJS is 1.2–6.0 per million, while the incidence of TEN is 0.4–1.2 per million annually [7], [8]. Both reactions are characterized by high mortality and morbidity rates despite the low incidence, as the mortality rate for SJS is 1–5% while for TEN it is 25–30 %[9,10]. SCARs are most commonly observed among patients taking carbamazepine (CBZ), phenytoin (PHT) and lamotrigine, and typically occur within the first three months of initiation [9].

SCARs, including SJS, TEN, and DRESS, are life-threatening conditions characterized by skin and mucosal lesions, with high risks of systemic complications. SJS/TEN leads to significant skin detachment, while DRESS involves a multi-organ response with skin rash, eosinophilia, and organ damage [11], [12,13].

The pathomechanism of SJS/TEN involves immune-driven keratinocyte apoptosis, primarily mediated by cytotoxic T-lymphocytes (CTLs) [14]. These CTLs trigger cell death through three main pathways: Fas-Fas ligand interaction, perforin-granzyme B, and granulysin, with granulysin being the most destructive, causing widespread keratinocyte necrosis and epidermal detachment[15,16] High levels of granulysin and soluble Fas ligand (sFasL) have been detected in the blister fluid of patients, both contributing to the immune response[17]. T-cells also produce granzyme B, inducing drug-specific cytotoxicity. The presence of granulysin in both blood and blister fluid suggests it could be a biomarker for predicting SJS/TEN prognosis. This immune response is mediated by *HLA*-restricted drug presentation, leading to the expansion of CD8^+^ T-lymphocytes, which are responsible for attacking keratinocytes [18], [19].

In 2004, Chung et al. reported a very strong association between carbamazepine-induced Stevens-Johnson Syndrome (SJS) and the *HLA-B∗15:02* allele in Han Chinese patients[20]. This landmark study primarily focused on SJS and did not explore other SCARs associated with carbamazepine. The U.S. Food and Drug Administration (FDA) and the Clinical Pharmacogenetics Implementation Consortium (CPIC) now recommend screening for the *HLA-B∗15:02* allele before initiating carbamazepine treatment in patients of Asian ancestry. Further studies, including a systematic review and meta-analysis, confirmed the strong association between the *HLA-B∗15:02* allele and carbamazepine-induced SJS and TEN across populations in Han Chinese, Thai, and Malaysian groups [21]. Additionally, Grover and Kukreti, in their meta-analysis, which showed a significant association between carbamazepine-induced SJS/TEN and both the *HLA*-*B∗15:02* and *HLA-B∗15:11* alleles among Asian population [22]. Interestingly, the *HLA-A∗31:01* allele was found to be associated with a broader spectrum of carbamazepine-induced hypersensitivity reactions, including maculopapular eruption (MPE) and DRESS, especially among patients of European descent [23].The *HLA-A∗31:01* allele has also been identified as a genetic predictor of carbamazepine-induced DRESS in Chinese and European populations, though it does not appear to play a significant role in SJS/TEN in these groups[24]. A separate study in Han Chinese patients highlighted an association between MPE/DRESS and both the *HLA-A∗31:01* and *HLA-B∗51:01* alleles [25].

This study aims to determine the association between *HLA-A, HLA-B, and HLA-DRB1* genotypes and the risk of AED-induced SCARs among case with AED-induced SCARs and AED-tolerant controls, the findings will enhance personalized medicine approaches and improve the safety of AED treatments.

## 2 Methodology

A total number of 50 adult patients (32 males with a mean age of 42.78 ± 12.82 years and 18 women with a mean age of 43.88 ± 14.78 years), and 90 tolerant controls (40 males with a mean age of 37.07 ± 14.10 years and 50 women with a mean age of 41.26 ± 16.15 years),living in the city of Baghdad, Capital of Iraq, participated in this study.

### 2.1 Patient selection and study criteria

Fifty patients were recruited in this study from 2020 to 2023, all of whom were diagnosed with SCAR diagnosis at the Dr. Saad Al-Wattari Hospital for Neurological Sciences and Baghdad Hospital - Medical City. The inclusion criteria comprised individuals diagnosed with SCARs such as SJS, TEN, or DRESS following treatment with AEDs like carbamazepine (CBZ), phenytoin (PHT), or lamotrigine (LTG). The control group included individuals treated with AEDs for at least three months without developing SCARs or other severe adverse reactions. Individuals with a history of SCARs associated with medications other than AEDs were excluded.

Demographic data, past medical history, and a list of drugs taken before admission were recorded for each patient, and SCARs were categorized into three main types: DRESS, SJS, and TEN.

Informed consent was obtained from all participants or their legal guardians before the commencement of the study, with clear explanations of the study's objectives and procedures. After informed consent was obtained, patients were interviewed by one of the authors. Strict measures were taken to ensure patient confidentiality, including the anonymization of personal data and secure storage of sensitive information.

### 2.2 HLA-A -B and DRB1 genotyping

Peripheral blood samples were collected for genotyping analysis. Genomic DNA was isolated using the ReliaPrepTM Blood gDNA Miniprep System Kit (Promega-USA) and stored at −20 °C until processing. DNA purity and concentration were measured using a Nanodrop UV spectrophotometer, with 1.5 μL of each DNA sample analyzed at absorbance wavelengths of 260 nm and 280 nm. Most samples had an A260/A280 ratio between 1.8 and 2.0, indicating good DNA purity suitable for further analysis, such as *HLA* gene polymorphism studies. DNA concentration ranged from 40 to 120 ng/μL for the majority of samples. DNA integrity was assessed using 1 % agarose gel electrophoresis. After electrophoresis at 100 V for 30 min, the gel was visualized under the UV light.

*HLA* typing was performed by using the LIFECODES *HLA* rSSO typing kit (Immucor, Stamford, CT, USA). PCR mixture was prepared with 6 μl of the LIFECODES Master Mix (Immucor), 4 μl of genomic DNA, and 0.2 U Taq polymerase and Nuclease Free water Up to 20 μl and then treated with the following: denaturation at 95 °C for 5 min; 40 cycles of amplification (12 cycles: 95 °C for 15 s, 60 °C for 30 s, 72 °C for 30 s, and 28 cycles: 95 °C for 10 s, 63 °C for 30 s, 72 °C for 30sec); and extension at 72 °C for 2 min. The reaction concluded by cooling to 4 °C in a final cycle.

Hybridization was performed under the following conditions: 97 °C for 2 min, 47 °C for 10 min, and 56 °C for 8 min with 15 μL probe mix and 5 μL of the PCR product. The samples were diluted with 170 μL of the pre-diluted streptavidin-phycoerythrin solution and analyzed within 30 min by using the Luminex 200 system (Luminex Corp.). The probe hit pattern was compared with the common and well-documented (CWD) *HLA* alleles Probe Hit Tables (IMGT/HLA Sequence Database Release 3.52.0) by using the MatchIT DNA program (Immucor).

### 2.3 Statistical analysis

SPSS version 15.0 was used for doing statistical analysis. (IBM, Chicago, Illinois, USA), Means and standard deviations (SD) were computed for all continuous data. The *t*-test was used to analyze the comparison of continuous variables between patients and controls based on clinical features. The comparison of *HLA-A, HLA-B*, and *HLA-DRB1* allele frequencies in each group was conducted using the chi-square test and Fisher's exact test. Univariate logistic regression was used to determine the odds ratio (OR) and 95 % confidence interval (CI) values. Sensitivity, specificity, positive predictive value (PPV), and negative predictive value (NPV) were calculated. The corrected P-values (*Pc*) for the multiple comparisons of *HLA* alleles were calculated using Bonferroni's correction. The statistical significance threshold was established at p < 0.05 (two-tailed).

### 2.4 Ethical approval

Ethical approval was obtained from the ethics board committee of Al-Nahrain University, Forensic DNA Research and Training Center, with approval number 116. Additionally, ethical approval was also obtained from the Baghdad Medical City, Ministry of Health of Iraq with approval number 1157 and Dr. Saad Al Watri Hospital with approval number 81595.

## 3 Results

**Fig. 1:**
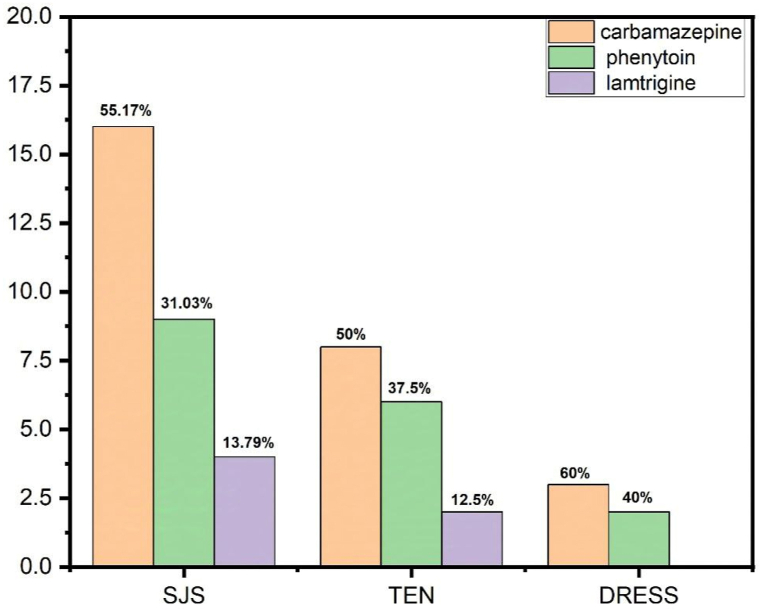
Types of SCARs and causing AEDs.

The clinical characteristics of patients with AED-induced cutaneous adverse drug reactions and AED-tolerant controls are detailed in Table 1. In the patient group, the causative antiepileptic drugs (AEDs) included CBZ (54 %), PHT (34 %), and LTG (12 %). In the tolerant control group, the AEDs were CBZ (54.4 %), PHT (17.7 %), and LTG (27.7 %). Overall, 50 cases of AEDs-induced SCARs were 29 SJS, 16 TEN and 5 DRESS cases. Specifically, CBZ was the leading cause in all three types of SCARs, accounting for over half of the SJS and TEN cases and the majority of DRESS cases. PHT was also with SJS and TEN, while LTG was less frequently implicated (see Fig. 1).

**Table 1:** Clinical characteristic of patients with AED-induced cutaneous adverse drug reactions and AED-tolerant controls.

Demographic Data	Case (n = 50)	Tolerant Control (n = 90)	P Value
**Gender (n/%)**			0.009
Male	32 (64 %)	37 (41.11 %)	
Female	18 (36 %)	53 (58.88 %)	
**Age (mean/range)**	43.18/16-70	39.4/17-71	0.15
**Type of epilepsy**	50 (100 %)	90 (100 %)	
Focal With Impaired Awareness	9/50	21/90	<0.001
Unknown Onset	2/50	–	<0.001
Generalized (Non-Motor)	5/50	23/90	<0.001
Focal Without Impaired Awareness	4/50	25/90	<0.001
Generalized (Motor)	17/50	21/90	<0.001
Complex Partial Seizures	6/50	–	<0.001
Absence	1/50	–	<0.001
Myoclonic Seizures	1/50	–	<0.001
Tonic-Clonic Seizures	3/50	–	<0.001
Partial Seizures	2/50	–	<0.001
**Other comorbidities**			
Anemia	–	1 (1.11 %)	0.45
Anxiety	–	2 (2.2 %)	0.15
Asthma	2 (4 %)	–	0.15
Autism spectrum disorders	–	1 (1.1 %)	0.45
breast cancer	–	2(2.2 %)	0.15
Chronic Lymphocytic Leukemia	–	1(1.11 %)	0.45
Chronic Rhinosinusitis	–	1(1.1 %)	0.45
Depression	1 (2 %)	2(2.2 %)	0.93
Diabetes	4 (8 %)	4 (4.4 %)	0.38
Gastritis	2 (4 %)	1(1.11 %)	0.34
Hyperlipidemia	–	1(1.11 %)	0.45
Hypertension	6 (12 %)	9 (10 %)	0.71
Hypertension, Diabetes	1 (2 %)	–	0.32
Hypothyroidism	–	2(2.2 %)	0.15
Nephrolithiasis	–	2(2.2 %)	0.15
Ocular Hypertension	–	1(1.11 %)	0.45
polycystic Ovary Syndrome	–	1(1.11 %)	0.45
prostate hypertrophy	1 (2 %)	–	0.32
Prostatitis	–	2(2.2 %)	0.15
Thrombocytopenia	–	1(1.11 %)	0.45
**Onset of cADRs (mean day/SD)**	14.48 ± 17.22	–	–
**Types and doses of AED**			
Carbamazepine	27 (54 %)	49(54.4 %)	0.96
Phenytoin	17(34 %)	16(17.7 %)	0.04
Lamotrigine	6(12 %)	25(27.7 %)	0.01
Dose of Carbamazepine, mg/day (mean ± SD)	407.40 ± 187.9	346.93 ± 214.18	<0.001
Dose of Phenytoin, mg/day (mean ± SD)	200 ± 86.60	229.41 ± 77.17	<0.001
Dose of Lamotrigine, mg/day (mean ± SD)	150 ± 54.77	90.740 ± 57.23	<0.001
**Types of SCAR**			
SJS	29/50	–	–
TEN	16/50	–	–
DRESS	5/50	–	–

The frequency of *HLA-A, B* and *DRB1* alleles in patients with SCARs, compared to tolerance controls is presented in Table 2, Table 3, Table 4, respectively. Table 5 provides a comprehensive summary of *HLA* allele frequencies in patients experiencing adverse drug reactions compared to a control group. The table includes various alleles, the number of frequencies in patients and controls, and the type of adverse reaction observed.

**Table 2:** The association between *HLA-A* alleles and the risk of AED-induced SCARs among Iraqi population.

*HLA-A* alleles	Patients	Control	P value	OR	95 % CI
*A∗01:01*	11	20	0.97	0.98	0.42–2.27
***A∗01:02***	**6**	**1**	**0.008**	**12.13**	**1.417–103.93**
***A∗02:01***	**13**	**39**	**0.04**	**0.45**	**0.21–0.97**
*A∗02:02*	3	1	0.13	5.68	0.57–56.13
*A∗02:05*	0	1	1.00	0.64	0.56–0.72
*A∗03:01*	11	11	0.12	2.02	0.80–5.08
*A∗03:02*	2	8	0.49	0.42	0.08–2.09
*A∗11:01*	3	10	0.37	0.51	0.13–1.94
*A∗11:02*	0	1	1.00	0.64	0.56–0.72
*A∗23:01*	4	15	0.15	0.43	0.13–1.39
*A∗24:01*	7	6	0.22	2.27	0.72–7.20
*A∗24:02*	9	7	0.06	2.60	0.90–7.48
*A∗26:01*	4	7	1.00	1.03	0.28–3.70
*A∗29:01*	2	5	1.00	0.70	0.13–3.79
*A∗29:02*	1	0	0.35	0.35	0.28–0.44
*A∗30:01*	4	7	1.00	1.03	0.28–3.70
***A∗30:02***	**3**	**0**	**0.04**	**0.34**	**0.27–0.43**
*A∗31:01*	0	1	1.00	0.64	0.56–0.72
*A∗31:03*	2	0	0.12	0.34	0.27–0.43
*A∗32:01*	4	8	1.00	0.89	0.25–3.12
*A∗32:02*	1	0	0.35	0.35	0.28–0.44
*A∗33:01*	2	10	0.21	0.33	0.07–1.58
*A∗33:03*	0	2	0.53	0.63	0.56–0.72
*A∗34:02*	1	0	0.35	0.35	0.28–0.44
*A∗36:01*	0	1	1.00	0.64	0.56–0.72
*A∗66:01*	1	0	0.35	0.35	0.28–0.44
*A∗68:01*	1	7	0.25	0.24	0.02–2.02
*A∗68:02*	0	2	0.53	0.63	0.56–0.72
*A∗69:01*	1	1	1.00	1.81	0.11–29.67

**Table 3:** The association between *HLA-B* alleles and the risk of AED-induced SCARs among Iraqi population.

*HLA-B* alleles	Patients	Control	P value	OR	95 % CI
*B∗07:01*	1	5	0.42	0.34	0.03–3.05
*B∗07:02*	1	8	0.15	0.20	0.02–1.72
*B∗07:05*	0	2	0.53	0.63	0.56–0.72
*B∗08:01*	3	5	1.00	1.08	0.24–4.74
*B∗08:02*	0	3	0.55	0.63	0.55–0.72
*B∗08:27*	1	3	1.00	0.59	0.05–5.84
*B∗13:01*	1	4	0.65	0.43	0.04–4.03
*B∗13:02*	0	2	0.53	0.63	0.56–0.72
*B∗14:01*	1	0	0.35	0.35	0.28–0.44
*B∗14:02*	1	6	0.42	0.28	0.03–2.44
*B∗15:01*	1	2	1.00	0.89	0.07–10.15
***B∗15:02***	**9**	**1**	**0.0004**	**19.53**	**2.39**–**159.35**
*B∗15:03*	1	5	0.42	0.34	0.03–3.05
*B∗18:01*	3	1	0.13	5.68	0.57–56.13
*B∗18:02*	0	3	0.55	0.63	0.55–0.72
*B∗27:01*	0	4	0.29	0.63	0.55–0.71
*B∗27:02*	1	0	0.35	0.35	0.28–0.44
*B∗35:01*	5	12	0.56	0.72	0.23–2.18
*B∗35:02*	3	8	0.74	0.65	0.16–2.58
*B∗35:03*	0	4	0.29	0.63	0.55–0.71
*B∗37:01*	1	0	0.35	0.35	0.28–0.44
*B∗38:01*	5	2	0.09	4.88	0.91–26.19
*B∗38:02*	0	2	0.53	0.63	0.56–0.72
*B∗39:01*	1	1	1.00	1.81	0.11–29.67
*B∗39:02*	0	1	1.00	0.64	0.56–0.72
*B∗40:01*	6	4	0.16	2.93	0.78–10.93
*B∗40:02*	4	1	0.05	7.73	0.84–71.25
*B∗40:04*	1	3	1.00	0.59	0.05–5.84
*B∗40:06*	0	3	0.55	0.63	0.55–0.72
*B∗41:01*	2	10	0.21	0.33	0.07–1.58
*B∗41:02*	0	3	0.55	0.63	0.55–0.72
*B∗42:01*	0	1	1.00	0.64	0.56–0.72
*B∗42:02*	1	1	1.00	1.81	0.11–29.67
*B∗44:01*	1	3	1.00	0.59	0.05–5.84
*B∗44:02*	1	7	0.25	0.24	0.02–2.02
*B∗44:03*	1	3	1.00	0.59	0.05–5.84
*B∗49:01*	1	9	0.09	0.18	0.02–1.49
*B∗49:02*	1	2	1.00	0.89	0.07–10.15
*B∗50:01*	2	6	0.71	0.58	0.11–3.00
*B∗50:02*	3	5	1.00	1.08	0.24–4.74
*B∗50:12*	0	5	0.16	0.62	0.55–0.71
*B∗51:01*	5	6	0.52	1.55	0.44–5.37
***B∗51:02***	**3**	**0**	**0.04**	**0.34**	**0.27–0.43**
*B∗51:04*	3	1	0.13	5.68	0.57–56.13
*B∗51:51*	0	1	1.00	0.64	0.56–0.72
*B∗52:01*	4	3	0.24	2.52	0.54–11.75
*B∗52:02*	0	2	0.53	0.63	0.56–0.72
*B∗53:01*	1	1	1.00	1.81	0.11–29.67
*B∗55:01*	1	3	1.00	0.59	0.05–5.84
*B∗57:01*	2	0	0.12	0.34	0.27–0.43
*B∗58:01*	2	2	0.61	1.83	0.25–13.42
*B∗58:02*	2	0	0.12	0.34	0.27–0.43
*B∗73:01*	1	0	0.35	0.35	0.28–0.44

**Table 4:** The association between *HLA-DRB1* alleles and the risk of AED-induced SCARs among Iraqi population.

*HLA-DRB1* alleles	PATIENTS	CONTROL	P value	OR	95 % CI
*DRB1∗01:01*	3	5	1.00	1.08	0.24–4.74
*DRB1∗01:02*	1	2	1.00	0.89	0.07–10.15
*DRB1∗01:03*	0	2	0.53	0.63	0.56–0.72
***DRB1∗03:01***	**18**	**16**	**0.01**	**2.60**	**1.17–5.73**
*DRB1∗03:02*	3	6	1.00	0.89	0.21–3.73
*DRB1∗03:03*	1	2	1.00	0.89	0.07–10.15
*DRB1∗04:01*	2	6	0.71	0.58	0.11–3.00
*DRB1∗04:02*	0	5	0.16	0.62	0.55–0.71
*DRB1∗04:03*	0	6	0.08	0.62	0.55–0.71
*DRB1∗04:06*	0	1	1.00	0.64	0.56–0.72
*DRB1∗04:14*	2	2	0.61	1.83	0.25–13.42
*DRB1∗04:145*	0	1	1.00	0.64	0.56–0.72
*DRB1∗07:01*	18	32	0.95	1.01	0.49–2.09
*DRB1∗07:02*	3	2	0.34	2.80	0.45–17.40
*DRB1∗08:01*	0	1	1.00	0.64	0.56–0.72
*DRB1∗08:04*	1	0	0.35	0.35	0.28–0.44
*DRB1∗09:01*	1	3	1.00	0.59	0.05–5.84
*DRB1∗10:01*	2	3	1.00	1.20	0.19–7.48
*DRB1∗10:02*	0	2	0.53	0.63	0.56–0.72
*DRB1∗10:12*	1	1	1.00	1.81	0.11–29.67
*DRB1∗11:01*	8	18	0.55	0.76	0.30–1.90
*DRB1∗11:02*	0	5	0.16	0.62	0.55–0.71
*DRB1∗11:03*	1	1	1.00	1.81	0.11–29.67
*DRB1∗11:04*	0	1	1.00	0.64	0.56–0.72
*DRB1∗11:06*	0	3	0.55	0.63	0.55–0.72
*DRB1∗11:37*	1	1	1.00	1.81	0.11–29.67
*DRB1∗11:48*	0	1	1.00	0.64	0.56–0.72
*DRB1∗11:50*	2	2	0.61	1.83	0.25–13.42
*DRB1∗11:74*	1	0	0.35	0.35	0.28–0.44
*DRB1∗12:01*	0	1	1.00	0.64	0.56–0.72
***DRB1∗13:01***	**13**	**8**	**0.006**	**3.60**	**1.37**–**9.42**
*DRB1∗13:02*	2	2	0.61	1.83	0.25–13.42
*DRB1∗13:07*	1	3	1.00	0.59	0.05–5.84
*DRB1∗13:256*	1	0	0.35	0.35	0.28–0.44
*DRB1∗13:40*	1	0	0.35	0.35	0.28–0.44
*DRB1∗13:70*	0	1	1.00	0.64	0.56–0.72
*DRB1∗14:01*	0	1	1.00	0.64	0.56–0.72
*DRB1∗14:02*	0	4	0.29	0.63	0.55–0.71
***DRB1∗15:01***	**9**	**6**	**0.03**	**3.07**	**1.02**–**9.21**
*DRB1∗15:02*	0	4	0.29	0.63	0.55–0.71
*DRB1∗15:03*	0	1	1.00	0.64	0.56–0.72
*DRB1∗15:04*	1	0	0.35	0.35	0.28–0.44
*DRB1∗15:07*	0	4	0.29	0.63	0.55–0.71
*DRB1∗16:01*	0	2	0.53	0.63	0.56–0.72

**Table 5:** Summary of significant *HLA* allele frequencies in patients with adverse reactions compared to control group.

*HLA* allele	Patients	Control	Adverse Reactions	P-Value	(OR)	95 % CI	*Pc*-value
*A∗02:01*	6	39	SJS	0.03	0.36	0.13–0.97	N.S
*A∗24:02*	7	7	SJS	0.02	3.60	1.21–10.72	N.S
*A∗31:03*	2	0	SJS	0.04	0.19	0.13–0.27	N.S
*B∗15:02*	5	1	SJS	0.03	4.41	1.18–16.47	N.S
*DRB1∗13:01*	9	8	SJS	0.01	3.71	1.38–9.97	N.S
*A∗01:02*	3	1	TEN	0.03	6.92	1.39–34.37	N.S
*B∗15:02*	4	1	TEN	0.01	6.55	1.62–26.52	N.S
*B∗52:01*	3	3	TEN	0.03	6.92	1.39–34.37	N.S
*DRB1∗03:01*	9	16	TEN	0.003	5.09	1.72–15.00	Sig.
*B∗40:02*	2	1	DRESS	0.009	29.33	3.50–245.32	N.S

Analysis of *HLA* allele frequencies revealed significant associations between specific alleles *HLA-A∗02:01* was found to be significantly associated with a lower risk of AED-induced SJS (p = 0.03; OR = 0.36; 95 % CI: 0.13–0.97), while *HLA-A∗24:02* and *HLA-B∗15:02* were associated with an increased risk of AED-induced SJS (p = 0.02; OR = 3.60; 95 % CI: 1.21–10.72 and p = 0.03; OR = 4.41; 95 % CI: 1.18–16.47, respectively).

For AED-induced TEN, *HLA-A∗01:02, HLA-B∗15:02*, and *HLA-B∗52:01* showed significant associations (p = 0.03; OR = 6.92; 95 % CI: 1.39–34.37 and p = 0.01; OR = 6.55; 95 % CI: 1.62–26.52, respectively), with *HLA-DRB1∗03:01* being highly significant (p = 0.003; OR = 5.09; 95 % CI: 1.72–15.00). Additionally, *HLA-B∗40:02* was strongly associated with AED-induced DRESS (p = 0.009; OR = 29.33; 95 % CI: 3.50–245.32).

The diagnostic performance of *HLA* alleles in predicting AED-induced SCARs was evaluated using sensitivity, specificity, positive predictive value (PPV), and negative predictive value (NPV). Table 6 summarizes these metrics, comparing the results between the AED-induced SCARs group and the tolerant control group.

**Table 6:** Sensitivity, Specificity, PPV, and NPV between AED-induced SCARs and tolerant control.

*HLA* allele	SJS				TEN				DRESS			
	Sensitivity	Specificity	PPV	NPV	Sensitivity	Specificity	PPV	NPV	Sensitivity	Specificity	PPV	NPV
*A∗02:01*	12	57	13	54	–	–	–	–	–	–	–	–
*A∗24:02*	14	92	50	66	–	–	–	–	–	–	–	–
*A∗31:03*	4	100	100	65	–	–	–	–	–	–	–	–
*B∗15:02*	10	99	83	66	8	99	80	66	–	–	–	–
*DRB1∗13:01*	18	91	53	67	–	–	–	–	–	–	–	–
*A∗01:02*	–	–	–	–	6	99	75	65	–	–	–	–
*B∗52:01*	–	–	–	–	6	97	50	65	–	–	–	–
*DRB1∗03:01*	–	–	–	–	18	82	36	64				
*B∗40:02*	–	–	–	–	–	–	–	–	4	99	67	

The prevalence of *HLA* alleles in patients treated with AEDs was analyzed across the *HLA-A, HLA-B, and HLA-DRB1* loci. Table 7 presents the distribution of *HLA-A* alleles, while Table 8 shows the prevalence of *HLA-B* alleles, and Table 9 provides the prevalence of *HLA-DRB1* alleles in the study population.

**Table 7:** Prevalence of *HLA-A* alleles in Patients Treated Antiepileptic drugs.

*HLA-A* alleles	Carbamazepine n (%)	Phenytoin n (%)	Lamotrigine n (%)
*A∗01:01*	7 (14 %)	2 (4 %)	2(4 %)
*A∗01:02*	3 (6 %)	2 (4 %)	1 (2 %)
*A∗02:01*	10 (20 %)	4 (8 %)	0
*A∗02:02*	2 (4 %)	1 (2 %)	0
*A∗03:01*	6 (12 %)	3(6 %)	2 (4 %)
*A∗03:02*	1 (2 %)	1 (2 %)	0
*A∗11:01*	1 (2 %)	2 (4 %)	0
*A∗23:01*	3 (6 %)	2 (4 %)	0
*A∗24:01*	3 (6 %)	3 (6 %)	1 (2 %)
*A∗24:02*	6 (12 %)	1 (2 %)	2 (4 %)
*A∗26:01*	2 (4 %)	2 (4 %)	0
*A∗29:01*	1 (2 %)	1 (2 %)	0
*A∗29:02*	0	1 (2 %)	0
*A∗30:01*	4 (8 %)	0	0
*A∗30:02*	0	1 (2 %)	2 (4 %)
*A∗31:03*	1 (2 %)	2 (4 %)	0
*A∗32:01*	2 (4 %)	1 (2 %)	1 (2 %)
*A∗32:02*	0	1 (2 %)	0
*A∗33:01*	0	1(2 %)	1 (2 %)
*A∗34:02*	0	2 (4 %)	0
*A∗66:01*	1(2 %)	0	0
*A∗68:01*	1 (2 %)	0	0
*A∗69:01*	0	1 (2 %)	0

**Table 8:** Prevalence of *HLA-B* alleles in Patients Treated Antiepileptic drugs.

*HLA-B* alleles	Carbamazepine n (%)	Phenytoin n (%)	Lamotrigine n (%)
*B∗07:01*	0	0	1
*B∗07:02*	1(2 %)	0	0
*B∗08:01*	2(4 %)	1(2 %)	0
*B∗08:27*	0	1(2 %)	0
*B∗13:01*	1(2 %)	0	0
*B∗14:01*	1(2 %)	0	0
*B∗14:02*	0	0	1(2 %)
*B∗15:01*	0	1(2 %)	0
*B∗15:02*	6(12 %)	2(4 %)	1(2 %)
*B∗15:03*	1(2 %)	0	0
*B∗18:01*	1(2 %)	1(2 %)	1(2 %)
*B∗27:02*	1(2 %)	0	0
*B∗35:01*	4(8 %)	0	1(2 %)
*B∗35:02*	1(2 %)	1(2 %)	1(2 %)
*B∗37:01*	1(2 %)	0	0
*B∗38:01*	3(6 %)	2(4 %)	0
*B∗39:01*	0	1(2 %)	0
*B∗40:01*	3(6 %)	2(4 %)	1(2 %)
*B∗40:02*	3(6 %)	1(2 %)	0
*B∗40:04*	0	1(2 %)	0
*B∗41:01*	1(2 %)	1(2 %)	0
*B∗42:02*	1(2 %)	0	0
*B∗44:01*	1(2 %)	0	0
*B∗44:02*	0	1(2 %)	0
*B∗44:03*	0	1(2 %)	0
*B∗49:01*	1(2 %)	0	0
*B∗49:02*	0	1(2 %)	0
*B∗50:01*	1(2 %)	1(2 %)	0
*B∗50:02*	2(4 %)	0	1(2 %)
*B∗51:01*	3(6 %)	2(4 %)	0
*B∗51:02*	1(2 %)	2(4 %)	0
*B∗51:04*	1(2 %)	2(4 %)	0
*B∗52:01*	2(4 %)	2(4 %)	0
*B∗53:01*	0	0	1(2 %)
*B∗55:01*	0	1(2 %)	0
*B∗57:01*	2(4 %)	0	0
*B∗58:01*	2(4 %)	0	0
*B∗58:02*	1(2 %)	0	1(2 %)
*B∗73:01*	0	0	1(2 %)

**Table 9:** Prevalence of *HLA-DRB1* alleles in Patients Treated Antiepileptic drugs.

*HLA-DRB1* alleles	Carbamazepine n (%)	Phenytoin n (%)	Lamotrigine n (%)
*DRB1∗01:01*	1(2 %)	1(2 %)	1(2 %)
*DRB1∗01:02*	1(2 %)	0	0
*DRB1∗03:01*	7(14 %)	9(18 %)	2(4 %)
*DRB1∗03:02*	2(4 %)	1(2 %)	0
*DRB1∗03:03*	0	1(2 %)	0
*DRB1∗04:01*	1(2 %)	0	1(2 %)
*DRB1∗04:14*	2(4 %)	0	0
*DRB1∗07:01*	10(20 %)	7(14 %)	1(2 %)
*DRB1∗07:02*	1(2 %)	0	2(4 %)
*DRB1∗08:04*	1(2 %)	0	0
*DRB1∗09:01*	0	1(2 %)	0
*DRB1∗10:01*	1(2 %)	1(2 %)	0
*DRB1∗10:12*	0	0	1(2 %)
*DRB1∗11:01*	4(8 %)	2(4 %)	2(4 %)
*DRB1∗11:03*	1(2 %)	0	0
*DRB1∗11:37*	1(2 %)	0	0
*DRB1∗11:50*	1(2 %)	1(2 %)	0
*DRB1∗11:74*	1(2 %)	0	0
*DRB1∗13:01*	8(16 %)	4(8 %)	1(2 %)
*DRB1∗13:02*	1(2 %)	1(2 %)	0
*DRB1∗13:07*	0	1(2 %)	0
*DRB1∗13:256*	0	1(2 %)	0
*DRB1∗13:40*	1(2 %)	0	0
*DRB1∗15:01*	7(14 %)	1(2 %)	1(2 %)
*DRB1∗15:04*	1(2 %)	0	0

The prevalence of *HLA* haplotypes in patients with adverse reactions to antiepileptic drugs was compared to the control group. The results are summarized in Table 10, highlighting the differences in haplotype frequencies between the two groups. The detailed data on the association between *HLA-A, HLA-B, and HLA-DRB1* genes and AED-induced SJS-TEN in Iraqi patients is provided in Table S1–3 in the supplementary file.

**Table 10:** Prevalence of HLA haplotype in Patients with Adverse Reactions Compared to Control Group.

Haplotype	Case(freq)	Control(freq)	Chi2	Fisher's p	Pearson's p	OR [95 % CI]
*A∗02:01B∗50:02DRB1∗07:01*	1(0.012)	2(0.016)	0.074	1	0.785	0.716 [0.063–8.03]
*A∗02:01B∗41:01DRB1∗15:07*	0(0)	2(0.016)	1.403	0.513	0.236	NA
*A∗24:02B∗35:01DRB1∗07:01*	0(0)	2(0.016)	1.403	0.513	0.236	NA
*A∗02:01B∗51:01DRB1∗11:02*	0(0)	2(0.016)	1.403	0.513	0.236	NA
*A∗02:01B∗50:12DRB1∗07:01*	0(0)	3(0.025)	2.116	0.27	0.145	NA
*A∗03:01B∗15:03DRB1∗07:01*	1(0.012)	1(0.008)	0.067	1	0.794	1.444 [0.089–23.43]
*A∗02:01B∗40:01DRB1∗15:01*	1(0.012)	2(0.016)	0.074	1	0.785	0.716 [0.063–8.03]
*A∗23:01B∗50:02DRB1∗03:01*	0(0)	2(0.016)	1.403	0.513	0.236	NA
*A∗23:01B∗50:01DRB1∗03:01*	0(0)	2(0.016)	1.403	0.513	0.236	NA
*A∗01:01B∗13:01DRB1∗07:01*	1(0.012)	1(0.008)	0.067	1	0.794	1.444 [0.089–23.43]
*A∗24:01B∗35:01DRB1∗03:01*	0(0)	2(0.016)	1.403	0.513	0.236	NA
*A∗11:01B∗49:01DRB1∗13:01*	0(0)	2(0.016)	1.403	0.513	0.236	NA
*A∗01:01B∗35:02DRB1∗03:01*	1(0.012)	1(0.008)	0.067	1	0.794	1.444 [0.089–23.43]
*A∗02:01B∗07:01DRB1∗03:01*	0(0)	4(0.033)	2.836	0.145	0.092	NA
*A∗24:02B∗35:01DRB1∗13:01*	2(0.024)	0(0)	2.907	0.166	0.088	NA
*A∗02:02B∗50:01DRB1∗13:01*	2(0.024)	0(0)	2.907	0.166	0.088	NA
*A∗24:01B∗38:01DRB1∗07:01*	2(0.024)	0(0)	2.907	0.166	0.088	NA
*A∗03:01B∗15:02DRB1∗03:01*	4(0.048)	0(0)	5.873	0.027	0.015	NA
*A∗01:02B∗58:01DRB1∗07:01*	2(0.024)	0(0)	2.907	0.166	0.088	NA
*A∗01:01B∗15:02DRB1∗13:01*	2(0.024)	0(0)	2.907	0.166	0.088	NA
*A∗24:01B∗51:04DRB1∗11:01*	2(0.024)	0(0)	2.907	0.166	0.088	NA
*A∗02:01B∗40:01DRB1∗03:01*	2(0.024)	0(0)	2.907	0.166	0.088	NA
*A∗01:02B∗38:01DRB1∗13:01*	2(0.024)	0(0)	2.907	0.166	0.088	NA
*A∗02:01B∗41:01DRB1∗03:01*	2(0.024)	0(0)	2.907	0.166	0.088	NA

## 4 Discussion

This study recruited patients with AED-induced hypersensitivity reactions, such as, SJS, TEN and DRESS and AED-tolerant patients from Iraq. We found the association between *HLA* alleles (*HLA-A∗01:02; HLA-A∗02:01, HLA-B∗15:02, HLA-DRB1∗03:01;HLA-DRB1∗13:01 and HLA-DRB1∗15:01*) and AED-induced SCARs. These results suggest a higher susceptibility to SCARs in patients positive for these alleles. These results suggest a higher susceptibility to SCARs in patients positive for these alleles. different studies, have found that specific *HLA* alleles are significantly associated with severe cutaneous adverse reactions, such as SJS, TEN, and DRESS, induced by AEDs.

*HLA* molecules play a crucial role in the immune system by presenting peptides, including drug-derived peptides, to T cells. Variations in *HLA* alleles can alter the peptide-binding groove of the *HLA* molecule, affecting which peptides are presented and the subsequent immune response. For instance, *HLA-B∗15:02* has a unique peptide-binding motif that allows it to present carbamazepine-derived peptides, triggering cytotoxic T cell responses and leading to severe skin reactions[26,27].

In contrast, alleles such as *HLA-A∗01:01*; *HLA-A∗02:01*; *HLA-A∗11:01* and *B∗07:02* or *B∗41:01* and *HLA-DRB1∗04:03* and *DRB1∗15:07* which were more frequent in controls, may present a different set of peptides that do not trigger such adverse immune responses. This protective effect could be due to the presentation of peptides that induce tolerance rather than an immune attack.

*HLA-A* is a gene that encodes a protein part of the major histocompatibility complex (*MHC*) class I. These proteins are found on the surface of most cells and play a crucial role in the immune system by presenting endogenous peptides (those from within the cell) to cytotoxic T cells. This presentation helps the immune system recognize and respond to infected or malignant cells, as these cells often produce abnormal or foreign peptides that are displayed by *HLA-A* molecules Cruz-Tapias, Castiblanco et al., 2013^.^ The *HLA-A∗02:01* and *HLA-A∗30:02* indicate its role in presenting drug-related antigens to T cells, thus triggering an immune response[29]. Meanwhile, *HLA-A∗01:02* allele has not been previously associated with drug hypersensitivity reactions.

Similar to *HLA-A, HLA-B* is also a gene within the MHC class I complex. The *HLA-B* proteins present intracellular peptides to CD8^+^ T cells, which are essential for the immune system's ability to detect and destroy cells that are infected with viruses or have turned cancerous. *HLA-B* is highly polymorphic, which allows for a broad range of peptide presentations and contributes to the immune system's versatility in recognizing various pathogens Cruz-Tapias, Castiblanco et al., 2013. *HLA-B∗15:02* and *HLA-B∗51:02* is well-documented in its association with AED-induced Stevens-Johnson Syndrome (SJS) and toxic epidermal necrolysis (TEN), particularly in Han Chinese and Thai populations The presence of this allele significantly increases the risk of severe ADRs to carbamazepine [10,20,[30], [31], [32]].

*HLA-DRB1* is a gene that encodes a protein part of the MHC class II complex. These proteins are primarily expressed on the surface of antigen-presenting cells, such as dendritic cells, macrophages, and B cells. *HLA-DRB1* presents extracellular peptides (those from outside the cell) to CD4^+^ helper T cells, which are crucial for orchestrating the immune response. By presenting peptides derived from pathogens, *HLA-DRB1* helps initiate and regulate adaptive immunity [28]. *HLA-DRB1∗03:01*, *HLA-DRB1∗13:01* and *HLA-DRB1∗15:01* alleles have also been implicated in drug hypersensitivity. These alleles influence the presentation of drug-derived peptides to CD4^+^ T cells, leading to a robust immune response that manifests as SCARs. The identification of these alleles in our study aligns with their known role in modulating immune responses and drug metabolism [33].

Previous studies have reported different alleles contributing to SCARs in various populations. For example, *HLA-A∗31:01* was noted in the Han Chinese, Japanese and Korean population [23,24]; *HLA-A∗51:01* in Iran [29]; *HLA-B∗15:01* in Han Chinese [34]; *HLA-B∗15:08* in the Indian population [35]; *HLA-B∗15:11* in Japanese and Han populations [36]; *HLA-B∗15:13* in the Malay population [37]; and *HLA-DRB1∗12:02* in the Han Chinese population [38]. These alleles have been associated with AED-induced SJS, TEN, and DRESS.

We found the association between *HLA alleles (HLA-A∗01:02; HLA-A∗02:01, HLA-A∗24:02, HLA-A∗31:03 HLA-B∗15:02, HLA-B∗52:01, HLA-B∗40:02 HLA-DRB1∗03:01and HLA-DRB1∗13:01)* and AED-induced SCARs. These results suggest a higher susceptibility to SCARs in patients positive for these alleles. Different studies have found that specific *HLA* alleles are significantly associated with SCARs, such as SJS, TEN, and DRESS, induced by AEDs. In one study, the most frequently observed *HLA* alleles in the Iraqi population were reported by Ref. [39], which are *HLA-A∗02, HLA-B∗35, and HLA-B∗07*. Additionally, a study by Ad'hiah and colleagues identified *HLA-A∗03:01, HLA-B∗35, and HLA-DRB1∗11* as the most prevalent alleles in this population [40].

The genetic variability in *HLA-A, HLA-B, and HLA-DRB1* alleles among patients treated with AEDs such as LGT, PHT, and CBZ reveals significant implications for drug-induced adverse reactions. The present study identified a notable association between *HLA-B∗15:02* and SCARs, including SJS, TEN, and DRESS in CBZ-treated patients. This finding is consistent with previous research, particularly in Asian populations, where *HLA-B∗15:02* has been strongly linked to CBZ-induced SJS/TEN. For instance, a study conducted among Han Chinese patients reported a high prevalence of *HLA-B∗15:02* in individuals who developed SJS/TEN following CBZ treatment [41].

In our study, *HLA-A∗24:02* was associated with an increased risk of AED-induced SJS, yielding a P-value of 0.02 and an OR of 3.60 (95 % CI: 1.21–10.72). Our findings align with meta-analyses indicating that *HLA-A∗24:02* as a risk factor for cutaneous ADRs across various populations beyond southern Han Chinese. Unlike *HLA-B∗15:02,* which is strongly linked to CBZ-induced SJS/TEN in Southeast Asian populations, *HLA-A∗24:02* is more broadly prevalent, indicating a potential role in drug-induced SCARs across different ethnic groups, including in Iraq. The differences in allele prevalence highlight the necessity of population-specific screening strategies to better understand the genetic predispositions that contribute to severe drug reactions. However, the need for larger studies is crucial to validate the association of *HLA-A∗24:02* with SJS in the Iraqi cohort[20,42,43].

In addition, the study observed a higher frequency of *HLA-A∗02:01* and *HLA-A∗01:01* in CBZ-treated patients [44]. Although these alleles are not as extensively studied in the context of SCARs, some research suggests that *HLA-A∗02:01* may thus be related to a broader range of drug hypersensitivity reactions. Furthermore, we identified a significant prevalence of *HLA-DRB1∗03:01* and *HLA-DRB1∗07:01* allele in patients treated with PHT and CBZ suggesting a genetic susceptibility to SCARs [45]. Similar associations have been reported in other studies, with *HLA-DRB1∗03:01* implicated in PHT-induced SCARs in Europeans and *HLA-DRB1∗07:01* in several drug-induced hypersensitivity reactions [46], [47].

The discrepancies between our results and previous reports underscore the genetic variability across different populations. Identifying novel predictors in our study highlights the importance of population-specific research in understanding the genetic basis of ADRs. This variability also suggests that the genetic background significantly influences the likelihood of developing SCARs in response to specific drugs. However, defects in several gene loci cannot be the only reason for any SCARs. Recognizing the other non-genetic factors and the level of their association with each SCARs can help increase specificity. Overall, paving the way to a precision medicine approach, both genetic and non-genetic factors should be studied in depth. For example, the incidence of SJS among children on LTG treatment has been estimated to be as high as 1:100 compared to adults, which is 1:1000 [48]. Recent Thai research, on the contrary, has found no link between age and the occurrence of AED-induced ADRs [10,49]. However, female gender is found to be at high risk of developing ADRs [50].

In this study, we have presented the sensitivity, specificity, positive predictive value (PPV), and negative predictive value (NPV) for various *HLA* alleles associated with AED-induced SCARs. Notably, *HLA-A∗24:02* exhibited high specificity 92 % but low sensitivity 14 % for SJS, indicating its strong potential for ruling out SJS in unaffected individuals but limited use in identifying those at risk. *HLA-B∗15:02* demonstrated high specificity 99 % for both SJS and TEN, making it a reliable marker for excluding risk, though its low sensitivity SJS: 10 %, TEN: 8 % limits its ability to detect all affected individuals. *HLA-DRB1∗13:01* had the highest sensitivity 18 % for SJS, paired with high specificity 91 %, reflecting a broader yet still limited predictive capability. For TEN, *HLA-A∗01:02* and *HLA-B∗52:01* both had high specificity 99 % and 97 %, respectively, but their PPV and NPV were moderate, limiting their predictive power. For DRESS, *HLA-B∗40:02* had low sensitivity 4 % but a high specificity 99 %, indicating a rare association. Overall, these findings underscore the high specificity of certain alleles like *HLA-B∗15:02* and *HLA-A∗24:02*, useful for ruling out SCARs, but their low sensitivity highlights the need for more comprehensive genetic and clinical screening strategies.

The analysis of haplotype associations with antiepileptic drug-induced SCARs revealed several interesting findings. The haplotype *A∗03:01B∗15:02DRB1∗03:01* was significantly associated with SCARs, being present only in cases and not in controls (p = 0.027). This suggests that individuals carrying this haplotype may have an increased genetic susceptibility to SCARs when exposed to antiepileptic drugs. Other haplotypes such as *A∗24:02B∗35:01DRB1∗13:01* and *A∗01:01B∗15:02DRB1∗13:01* were also found exclusively in cases, and they did not reach statistical significance (p = 0.166). This could be due to the limited sample size, which may have reduced the power to detect stronger associations. Additionally, some haplotypes, such as *A∗02:01B∗50:02DRB1∗07:01* (p = 0.785, OR = 0.716), were present in both cases and controls without significant differences, indicating they are unlikely to be risk factors for SCARs in this population.

Our results emphasize the importance of *HLA* alleles as key predictors of severe complications from AED therapy, helping to identify individuals at higher risk and allowing for more personalized treatment. Screening for *HLA* alleles linked to AED-induced SCARs, including SJS, TEN, and DRESS, is crucial for improving patient safety and optimizing treatment in the Iraqi population, similar to approaches in East Asia. Expanding this screening in Iraq can uncover genetic risk factors, leading to better healthcare outcomes. Identifying specific HLA haplotypes will also guide strategies to reduce adverse drug reactions. Future studies with larger and more diverse groups are needed to explore these genetic predictors further.

## 5 Conclusion

Our study highlights significant associations between specific *HLA* alleles and the susceptibility to severe cutaneous adverse reactions (SCARs) induced by antiepileptic drugs (AEDs) in the Iraqi population. The alleles *HLA-A∗01:02*, *HLA-A∗02:01*, *HLA-B∗15:02*, *HLA-DRB1∗03:01*, *HLA-DRB1∗13:01*, and *HLA-DRB1∗15:01* were found to be significantly more frequent in patients with SCARs compared to AED-tolerant individuals. Notably, *HLA-A∗01:02*, which has not been previously associated with drug hypersensitivity reactions, emerged as a novel risk factor in this population.

These findings underscore the importance of population-specific genetic research in understanding the genetic basis of adverse drug reactions (ADRs). The identification of these associations may aid in the development of predictive genetic tests, enabling personalized medicine approaches to reduce the risk of SCARs in patients requiring AED treatment. Future studies with larger cohorts and diverse populations are essential to validate these associations and further elucidate the mechanisms underlying *HLA*-mediated drug hypersensitivity.

## 6 Limitations of the study and future directions

The limitations of the study include its focus on a single city, which may affect the generalizability of the findings to other regions, and as well as a relatively small sample size, which restricted the analysis of clinical outcomes and non-genetic factors. Future research with larger, multicenter cohorts and diverse populations is necessary to validate these findings and enhance understanding of HLA gene predictors in drug-induced SCARs.

Future research should include studies on diverse population groups within Iraq and genetically similar populations to validate the associations identified in this study. It is also essential to clarify the precise mechanisms by which certain HLA alleles contribute to SCARs in response to specific drugs, including how these alleles interact with drug-derived peptides to trigger immune responses and cause tissue damage. Additionally, developing standardized, cost-effective techniques for identifying and classifying HLA alleles will help stratify individuals most susceptible to SCARs, aiding in clinical decisions regarding drug selection and dosing before treatment. Through the above suggestions, precision medicine strategies are beneficial in the prevention of drug adverse reactions, ultimately leading to better patient outcomes and improved safety in clinical settings.

## CRediT authorship contribution statement

**Ali Fadhel Ahmed:** Writing – original draft, Methodology, Investigation, Formal analysis. **Dzul Azri Mohamed Noor:** Writing – review & editing, Supervision. **Majeed Arsheed Sabbah:** Writing – review & editing, Supervision, Resources, Methodology, Investigation. **Nur Fadhlina Musa:** Writing – review & editing, Methodology. **Nur Aizati Athirah Daud:** Writing – review & editing, Supervision, Conceptualization.

## Declaration of competing interest

The authors declare no conflict of interest in the preparation of this manuscript.
