# Supplementary material for: Pharmacogenomics predictors of aromatic antiepileptic drugs-induced SCARs in the Iraqi patients
Source: Heliyon. 2024 Dec 18;11(1):e41108. doi: 10.1016/j.heliyon.2024.e41108 (PMC11732454; doi:10.1016/j.heliyon.2024.e41108)
Supplement: Multimedia component 1 [file mmc1.docx]

**Pharmacogenomics Predictors of Aromatic Antiepileptic Drugs-induced SCARs in the Iraqi Patients**

Ali Fadhel Ahmed^1^ , , Dzul Azri Mohamed Noor^1^, Majeed Arsheed Sabbah^3^ and , Nur Fadhlina Musa^2^, Nur Aizati Athirah Daud^1,2, *^

1. Discipline of Clinical Pharmacy, School of Pharmaceutical Sciences, Universiti Sains Malaysia, 11800 USM Pulau Pinang, Malaysia; alifadhel1131990@student.usm.my or alifadhel1131990@gmail.com (A.F.A.); aizati@usm.my (N.A.A.D); dzulazri@usm.my (D.A.M.N.)
2. Human Genome Centre, School of Medical Sciences, Universiti Sains Malaysia Health Campus, 16150 Kubang Kerian, Kelantan, Malaysia; fadhlina@usm.my
3. Forensic DNA for Research and Training Centre, Alnahrain University, Baghdad 64074, Iraq; majeedbio@gmail.com

**Correspondence:** Nur Aizati Athirah Daud; aizati@usm.my or aizati.daud@gmail.com

**Table S1 The association between *HLA-A* in patients with Stevens–Johnson syndrome (SJS) compared to tolerance controls**

| **95% CI** | **(OR)** | **P-Value** | **CONTROL** | **PATIENTS** | ***HLA-A*** |
| --- | --- | --- | --- | --- | --- |
| S | 1.15 | 0.77 | 21 | 7 | *A*01:01* |
| 0.65-14.64 | 3.08 | 0.15 | 1 | 3 | *A*01:02* |
| **0.13 - 0.97** | **0.36** | **0.03** | **46** | **7** | ***A*02:01*** |
| 0.54-29.97 | 4.03 | 0.19 | 1 | 2 | *A*02:02* |
| 0.72-0.8 | 0.79 | 1.00 | 1 | 0 | *A*02:05* |
| 0.38-3.43 | 1.15 | 0.77 | 11 | 5 | *A*03:01* |
| 0.04-3.33 | 0.40 | 0.68 | 8 | 1 | *A*03:02* |
| 0.14-3.22 | 0.67 | 1.00 | 10 | 2 | *A*11:01* |
| 0.72-0.86 | 0.79 | 1.00 | 1 | 0 | *A*11:02* |
| 0.08-1.88 | 0.41 | 0.36 | 15 | 2 | *A*23:01* |
| 0.80-8.92 | 2.68 | 0.14 | 6 | 5 | *A*24:01* |
| **1.21-10.72** | **3.60** | **0.02** | **7** | **7** | ***A*24:02*** |
| 0.17-4.11 | 0.84 | 1.00 | 7 | 2 | *A*26:01* |
| 0.71-0.85 | 0.78 | 0.34 | 5 | 0 | *A*29:01* |
| 0.72-0.86 | 0.79 | 1.00 | 1 | 0 | *A*29:01* |
| 0.64-8.75 | 2.37 | 0.23 | 8 | 4 | *A*30:01* |
| 0.71-93.21 | 8.14 | 0.10 | 0 | 2 | *A*30:02* |
| 0.72-0.86 | 0.79 | 1.00 | 1 | 0 | *A*31:01* |
| **0.13-0.27** | **0.19** | **0.04** | **0** | **3** | ***A*31:03*** |
| 0.15-3.61 | 0.74 | 1.00 | 8 | 2 | *A*32:01* |
| 0.14-0.28 | 0.20 | 0.20 | 0 | 1 | *A*32:02* |
| 0.04-2.62 | 0.32 | 0.46 | 10 | 1 | *A*33:01* |
| 0.72-0.86 | 0.79 | 1.00 | 2 | 0 | *A*33:03* |
| 0.72-0.86 | 0.79 | 1.00 | 0 | 2 | *A*34:02* |
| 0.72-0.86 | 0.79 | 1.00 | 1 | 0 | *A*36:01* |
| 0.14-0.28 | 0.20 | 0.20 | 0 | 1 | *A*66:01* |
| 0.71-0.85 | 0.78 | 0.20 | 7 | 0 | *A*68:01* |
| 0.72-0.86 | 0.79 | 1.00 | 2 | 0 | *A*68:02* |
| 0.23-64.77 | 3.92 | 0.37 | 1 | 1 | *A*69:01* |

**Table S2 The association between *HLA-B* in patients with Stevens–Johnson syndrome (SJS) compared to tolerance controls**

| **CI95%** | **Od** | **P value** | CONTROL | PATIENTS | *HLA-B* |
| --- | --- | --- | --- | --- | --- |
| 0.08-6.74 | 0.75 | 1.00 | 6 | 1 | *B*07:01* |
| 0.71-0.85 | 0.77 | 0.20 | 10 | 0 | *B*07:02* |
| 0.72-0.86 | 0.79 | 1.00 | 2 | 0 | *B*07:05* |
| 0.24-6.78 | 1.29 | 0.67 | 5 | 2 | *B*08:01* |
| 0.72-0.86 | 0.78 | 1.00 | 4 | 0 | *B*08:02* |
| 0.12-12.83 | 1.28 | 1.00 | 3 | 1 | *B*08:27* |
| 0.10-8.88 | 0.95 | 1.00 | 4 | 2 | *B*13:01* |
| 0.72-0.86 | 0.79 | 1.00 | 2 | 0 | *B*13:02* |
| 0.14-0.28 | 0.20 | 0.20 | 0 | 1 | *B*14:01* |
| 0.07-5.40 | 0.62 | 1.00 | 6 | 1 | *B*14:02* |
| 0.17-22.24 | 1.94 | 0.50 | 2 | 1 | *B*15:01* |
| 1.18-16.47 | 4.41 | 0.03 | 1 | 5 | *B*15:02* |
| 0.08-6.74 | 0.75 | 1.00 | 5 | 1 | *B*15:03* |
| 0.02-4.01 | 0.33 | 1.00 | 1 | 1 | *B*18:01* |
| 0.72-0.86 | 0.78 | 1.00 | 3 | 0 | *B*18:02* |
| 0.72-0.85 | 0.78 | 0.58 | 4 | 0 | *B*27:01* |
| 0.44-0.72 | 0.57 | 0.20 | 0 | 1 | *B*27:02* |
| 0.55-5.34 | 1.71 | 0.34 | 12 | 6 | *B*35:01* |
| 0.36-5.99 | 1.48 | 0.69 | 8 | 3 | *B*35:02* |
| 0.72-0.85 | 0.78 | 0.58 | 4 | 0 | *B*35:03* |
| 0.14-0.28 | 0.20 | 0.20 | 0 | 1 | *B*37:01* |
| 0.28-8.53 | 1.57 | 0.63 | 2 | 2 | *B*38:01* |
| 0.72-0.86 | 0.79 | 1.00 | 2 | 0 | *B*38:02* |
| 0.23-64.77 | 3.92 | 0,37 | 1 | 1 | *B*39:01* |
| 0.72-0.86 | 0.79 | 1.00 | 2 | 0 | *B*39:02* |
| 0.41-7.08 | 1.71 | 0.43 | 4 | 2 | *B*40:01* |
| 0.10-8.88 | 0.95 | 1.00 | 1 | 2 | *B*40:02* |
| 0.12-12.83 | 1.28 | 1.00 | 4 | 2 | *B*40:04* |
| 0.72-0.86 | 0.78 | 1.00 | 3 | 0 | *B*40:06* |
| 0.04-2.62 | 0.32 | 0.46 | 10 | 1 | *B*41:01* |
| 0.72-0.86 | 0.78 | 1.00 | 3 | 0 | *B*41:02* |
| 0.72-0.86 | 0.79 | 1.00 | 1 | 0 | *B*42:01* |
| 0.23-64.77 | 3.92 | 0.37 | 1 | 1 | *B*42:02* |
| 0.12-12.83 | 1.28 | 1.00 | 3 | 1 | *B*44:01* |
| 0.06-4.49 | 0.53 | 1.00 | 8 | 1 | *B*44:02* |
| 0.72-0.85 | 0.78 | 0.58 | 3 | 0 | *B*44:03* |
| 0.04-3.33 | 0.40 | 0.68 | 11 | 1 | *B*49:01* |
| 0.17-22.24 | 1.94 | 0.50 | 2 | 1 | *B*49:02* |
| 0.06-4.49 | 0.53 | 1.00 | 6 | 1 | *B*50:01* |
| 0.06-4.49 | 0.53 | 1.00 | 5 | 1 | *B*50:02* |
| 0.71-0.85 | 0.78 | 0.58 | 5 | 0 | *B*50:12* |
| 0.06-2.93 | 0.44 | 1.00 | 8 | 2 | *B*51:01* |
| 0.17-22.24 | 1.94 | 0.50 | 0 | 1 | *B*51:02* |
| 0.54-29.97 | 4.03 | 0.19 | 1 | 2 | *B*51:04* |
| 0.72-0.86 | 0.79 | 1.00 | 1 | 0 | *B*51:51* |
| 0.07-5.40 | 0.62 | 1.00 | 3 | 1 | *B*52:01* |
| 0.72-0.86 | 0.79 | 1.00 | 2 | 0 | *B*52:02* |
| 0.23-64.77 | 3.92 | 0.37 | 1 | 1 | *B*53:01* |
| 0.12-12.83 | 1.28 | 1.00 | 3 | 1 | *B*55:01* |
| 0.23-64.77 | 3.92 | 0.37 | 0 | 1 | *B*57:01* |
| 0.12-12.83 | 1.28 | 1.00 | 2 | 1 | *B*58:01* |
| 0.23-64.77 | 3.92 | 0.37 | 0 | 1 | *B*58:02* |
| 0.14-0.28 | 0.20 | 0.20 | 0 | 1 | *B*73:01* |

**Table S3 The association between *HLA-DRB1* in patients with Stevens–Johnson syndrome (SJS) compared to tolerance controls**

| **CI95%** | **Od** | **P value** | **CONTROL** | **PATIENTS** | ***HLA-DRB1*** |
| --- | --- | --- | --- | --- | --- |
| 0.24-6.78 | 1.29 | 0.67 | 5 | 2 | *DRB1*01:01* |
| 0.72-0.86 | 0.78 | 1.00 | 2 | 0 | *DRB1*01:02* |
| 0.72-0.86 | 0.79 | 1.00 | 2 | 0 | *DRB1*01:03* |
| 0.49-3.14 | 1.24 | 0.80 | 20 | 8 | *DRB1*03:01* |
| 0.05-3.83 | 0.46 | 0.68 | 6 | 1 | *DRB1*03:02* |
| 0.72-0.86 | 0.78 | 1.00 | 3 | 0 | *DRB1*03:03* |
| 0.24-6.78 | 1.29 | 0.67 | 7 | 2 | *DRB1*04:01* |
| 0.71-0.85 | 0.78 | 0.58 | 5 | 0 | *DRB1*04:02* |
| 0.71-0.85 | 0.78 | 0.34 | 6 | 0 | *DRB1*04:03* |
| 0.72-0.86 | 0.79 | 1.00 | 2 | 0 | *DRB1*04:06* |
| 0.12-12.83 | 1.28 | 1.00 | 2 | 1 | *DRB1*04:14* |
| 0.72-0.86 | 0.79 | 1.00 | 1 | 0 | *DRB1*04:145* |
| 0.32-1.84 | 0.76 | 0.66 | 35 | 9 | *DRB1*07:01* |
| 0.99-39.58 | 6.28 | 0.06 | 2 | 3 | *DRB1*07:02* |
| 0.72-0.86 | 0.79 | 1.00 | 1 | 0 | *DRB1*08:01* |
| 0.14-0.28 | 0.20 | 0.20 | 0 | 1 | *DRB1*08:04* |
| 0.12-12.83 | 1.28 | 1.00 | 3 | 1 | *DRB1*09:01* |
| 0.10-8.88 | 0.95 | 1.00 | 3 | 1 | *DRB1*10:01* |
| 0.72-0.86 | 0.79 | 1.00 | 2 | 0 | *DRB1*10:02* |
| 0.23-64.77 | 3.92 | 0.37 | 1 | 1 | *DRB1*10:12* |
| 0.42-3.29 | 1.18 | 0.79 | 18 | 7 | *DRB1*11:01* |
| 0.71-0.85 | 0.78 | 0.58 | 7 | 0 | *DRB1*11:02* |
| 0.72-0.86 | 0.79 | 1.00 | 1 | 0 | *DRB1*11:03* |
| 0.72-0.86 | 0.79 | 1.00 | 1 | 0 | *DRB1*11:04* |
| 0.72-0.86 | 0.78 | 1.00 | 3 | 0 | *DRB1*11:06* |
| 0.72-0.86 | 0.79 | 1.00 | 1 | 0 | *DRB1*11:37* |
| 0.72-0.86 | 0.79 | 1.00 | 1 | 0 | *DRB1*11:48* |
| 0.54-29.97 | 4.03 | 0.19 | 2 | 2 | *DRB1*11:50* |
| 0.14-0.28 | 0.20 | 0.20 | 0 | 1 | *DRB1*11:74* |
| 0.72-0.86 | 0.79 | 1.00 | 1 | 0 | *DRB1*12:01* |
| 1.38-9.98 | 3.71 | 0.01 | 8 | 8 | *DRB1*13:01* |
| 0.12-12.83 | 1.28 | 1.00 | 2 | 2 | *DRB1*13:02* |
| 0.72-0.85 | 0.78 | 0.58 | 3 | 0 | *DRB1*13:07* |
| 0.14-0.28 | 0.20 | 0.20 | 0 | 1 | *DRB1*13:256* |
| 0.72-0.86 | 0.79 | 1.00 | 1 | 0 | *DRB1*13:70* |
| 0.72-0.86 | 0.79 | 1.00 | 1 | 0 | *DRB1*14:01* |
| 0.72-0.85 | 0.78 | 0.58 | 4 | 0 | *DRB1*14:02* |
| 0.95-9.13 | 2.95 | 0.08 | 6 | 7 | *DRB1*15:01* |
| 0.72-0.85 | 0.78 | 0.58 | 5 | 0 | *DRB1*15:02* |
| 0.72-0.86 | 0.79 | 1.00 | 1 | 0 | *DRB1*15:03* |
| 0.72-0.86 | 0.79 | 1.00 | 4 | 0 | *DRB1*15:07* |
| 0.72-0.85 | 0.78 | 0.58 | 2 | 0 | *DRB1*16:01* |

**Table S4 The association between *HLA-A* in patients with Toxic Epidermal Necrolysis (TEN), compared to tolerance controls**

| **CI95%** | **OR** | **P value** | **CONTROL** | **PATIENTS** | ***HLA-A*** |
| --- | --- | --- | --- | --- | --- |
| 0.35-4.01 | 1.19 | 0.75 | 21 | 4 | *A*01:01* |
| 1.39-34.38 | 6.92 | 0.03 | 1 | 3 | *A*01:02* |
| 0.24-2.27 | 0.74 | 0.60 | 46 | 5 | *A*02:01* |
| 0.83-0.93 | 0.88 | 1.00 | 1 | 0 | *A*02:02* |
| 0.83-0.94 | 0.88 | 1.00 | 1 | 0 | *A*02:05* |
| 0.88-9.25 | 2.86 | 0.13 | 11 | 5 | *A*03:01* |
| 0.10-7.20 | 0.85 | 1.00 | 8 | 1 | *A*03:02* |
| 0.07-5.13 | 0.62 | 1.00 | 10 | 1 | *A*11:01* |
| 0.83-0.94 | 0.88 | 1.00 | 1 | 0 | *A*11:02* |
| 0.80-0.93 | 0.86 | 0.12 | 15 | 0 | *A*23:01* |
| 0.07-5.13 | 0.62 | 1.00 | 6 | 1 | *A*24:01* |
| 0.06-3.93 | 0.48 | 0.69 | 7 | 1 | *A*24:02* |
| 0.09-6.36 | 0.76 | 1.00 | 7 | 1 | *A*26:01* |
| 0.60-19.19 | 3.40 | 0.18 | 5 | 2 | *A*29:01* |
| 0.06-0.17 | 0.10 | 0.11 | 0 | 1 | *A*29:02* |
| 0.82-0.93 | 0.87 | 0.36 | 8 | 0 | *A*30:01* |
| 0.34-47.58 | 4.06 | 0.30 | 0 | 1 | *A*30:02* |
| 0.83-0.94 | 0.88 | 1.00 | 1 | 0 | *A*31:01* |
| 0.32-8.20 | 1.62 | 0.62 | 8 | 2 | *A*32:01* |
| 0.83-0.94 | 0.88 | 1.00 | 0 | 0 | *A*32:02* |
| 0.08-5.68 | 0.68 | 1.00 | 10 | 1 | *A*33:01* |
| 0.83-0.93 | 0.88 | 1.00 | 2 | 0 | *A*33:03* |
| 0.06-0.17 | 0.10 | 0.11 | 0 | 2 | *A*34:02* |
| 0.83-0.94 | 0.88 | 1.00 | 1 | 0 | *A*36:01* |
| 0.12-9.62 | 1.11 | 1.00 | 7 | 1 | *A*68:01* |
| 0.83-0.93 | 0.88 | 1.00 | 2 | 0 | *A*68:02* |
| 0.83-0.93 | 0.88 | 1.00 | 1 | 0 | *A*69:01* |

**Table S5 The association between *HLA-B* in patients with Toxic Epidermal Necrolysis (TEN), compared to tolerance controls**

| **CI95%** | **OR** | **P value** | CONTROL | PATIENTS | *HLA-B* |
| --- | --- | --- | --- | --- | --- |
| 0.82-0.93 | 0.88 | 1.00 | 6 | 0 | *B*07:01* |
| 0.82-0.93 | 0.87 | 0.59 | 10 | 0 | *B*07:02* |
| 0.83-0.93 | 0.88 | 1.00 | 2 | 0 | *B*07:05* |
| 0.82-0.93 | 0.87 | 0.59 | 5 | 0 | *B*08:01* |
| 0.83-0.93 | 0.88 | 1.00 | 4 | 0 | *B*08:02* |
| 0.83-0.93 | 0.88 | 1.00 | 3 | 0 | *B*08:27* |
| 0.82-0.93 | 0.88 | 1.00 | 4 | 0 | *B*13:01* |
| 0.83-0.93 | 0.88 | 1.00 | 2 | 0 | *B*13:02* |
| 0.82-0.93 | 0.88 | 1.00 | 6 | 0 | *B*14:02* |
| 0.83-0.93 | 0.88 | 1.00 | 2 | 0 | *B*15:01* |
| 1.62-26.52 | 6.55 | 0.01 | 1 | 5 | *B*15:02* |
| 0.82-0.93 | 0.88 | 1.00 | 5 | 0 | *B*15:03* |
| 0.39-56.32 | 4.71 | 0.06 | 1 | 2 | *B*18:01* |
| 0.83-0.93 | 0.88 | 1.00 | 3 | 0 | *B*18:02* |
| 0.83-0.93 | 0.88 | 1.00 | 4 | 0 | *B*27:01* |
| 0.81-0.93 | 0.87 | 0.21 | 12 | 0 | *B*35:01* |
| 0.82-0.93 | 0.87 | 0.36 | 8 | 0 | *B*35:02* |
| 0.83-0.93 | 0.88 | 1.00 | 4 | 0 | *B*35:03* |
| 0.60-19.19 | 3.40 | 0.18 | 2 | 2 | *B*38:01* |
| 0.83-0.93 | 0.88 | 1.00 | 2 | 0 | *B*38:02* |
| 0.83-0.93 | 0.88 | 1.00 | 1 | 0 | *B*39:01* |
| 0.83-0.94 | 0.88 | 1.00 | 2 | 0 | *B*39:02* |
| 0.40-10.74 | 2.07 | 0.32 | 4 | 4 | *B*40:01* |
| 0.21 – 19.09 | 2 | 0.46 | 1 | 1 | *B*40:02* |
| 0.83-0.93 | 0.88 | 1.00 | 4 | 0 | *B*40:04* |
| 0.83-0.93 | 0.88 | 1.00 | 3 | 0 | *B*40:06* |
| 0.08-5.68 | 0.68 | 1.00 | 10 | 1 | *B*41:01* |
| 0.83-0.93 | 0.88 | 1.00 | 3 | 0 | *B*41:02* |
| 0.83-0.94 | 0.88 | 1.00 | 1 | 0 | *B*42:01* |
| 0.83-0.93 | 0.88 | 1.00 | 1 | 0 | *B*42:02* |
| 0.83-0.93 | 0.88 | 1.00 | 3 | 0 | *B*44:01* |
| 0.82-0.93 | 0.87 | 0.59 | 8 | 0 | *B*44:02* |
| 0.26-27.52 | 2.68 | 0.38 | 3 | 2 | *B*44:03* |
| 0.82-0.93 | 0.87 | 0.60 | 11 | 0 | *B*49:01* |
| 0.83-0.93 | 0.88 | 1.00 | 2 | 0 | *B*49:02* |
| 0.82-0.93 | 0.87 | 0.59 | 6 | 0 | *B*50:01* |
| 0.12-9.69 | 1.11 | 1.00 | 5 | 1 | *B*50:02* |
| 0.82-0.93 | 0.87 | 0.59 | 5 | 0 | *B*50:12* |
| 0.78-14.20 | 3.34 | 0.11 | 8 | 3 | *B*51:01* |
| 1.49-206.34 | 17.57 | 0.03 | 0 | 2 | *B*51:02* |
| 0.26 – 27.52 | 2.68 | 0.38 | 1 | 1 | *B*51:04* |
| 0.83-0.94 | 0.88 | 1.00 | 1 | 0 | *B*51:51* |
| 1.39-34.38 | 6.92 | 0.03 | 3 | 3 | *B*52:01* |
| 0.83-0.93 | 0.88 | 1.00 | 2 | 0 | *B*52:02* |
| 0.83-0.93 | 0.88 | 1.00 | 1 | 0 | *B*53:01* |
| 0.83-0.93 | 0.88 | 1.00 | 3 | 0 | *B*55:01* |
| 0.48-138 | 8.20 | 0.21 | 0 | 1 | *B*57:01* |
| 0.26-27.52 | 2.68 | 0.38 | 2 | 1 | *B*58:01* |
| 0.48-138 | 8.20 | 0.21 | 0 | 1 | *B*58:02* |

**Table S6 The association between *HLA-DRB1* in patients with Toxic Epidermal Necrolysis (TEN), compared to tolerance controls**

| **CI95%** | **OR** | **P value** | **CONTROL** | **PATIENTS** | ***HLA-DRB1*** |
| --- | --- | --- | --- | --- | --- |
| 0.12-9.62 | 1.11 | 1.00 | 5 | 1 | *DRB1*01:01* |
| 0.34-47.58 | 4.06 | 0.30 | 2 | 1 | *DRB1*01:02* |
| 0.83-0.93 | 0.88 | 1.00 | 2 | 0 | *DRB1*01:03* |
| 1.72-15 | 5.09 | 0.004 | 20 | 10 | *DRB1*03:01* |
| 0.11-8.27 | 0.96 | 1.00 | 6 | 1 | *DRB1*03:02* |
| 0.83-0.93 | 0.88 | 1.00 | 3 | 0 | *DRB1*03:03* |
| 0.82-0.93 | 0.87 | 0.59 | 7 | 0 | *DRB1*04:01* |
| 0.82-0.93 | 0.88 | 1.00 | 5 | 0 | *DRB1*04:02* |
| 0.82-0.93 | 0.88 | 1.00 | 6 | 0 | *DRB1*04:03* |
| 0.83-0.94 | 0.88 | 1.00 | 2 | 0 | *DRB1*04:06* |
| 0.26-27.52 | 2.68 | 0.38 | 2 | 1 | *DRB1*04:14* |
| 0.83-0.94 | 0.88 | 1.00 | 1 | 0 | *DRB1*04:145* |
| 0.51-4.20 | 1.46 | 0.47 | 35 | 7 | *DRB1*07:01* |
| 0.82-0.93 | 0.88 | 1.00 | 2 | 0 | *DRB1*07:02* |
| 0.83-0.94 | 0.88 | 1.00 | 1 | 0 | *DRB1*08:01* |
| 0.83-0.93 | 0.88 | 1.00 | 3 | 0 | *DRB1*09:01* |
| 0.21-19.09 | 2 | 0.46 | 3 | 1 | *DRB1*10:01* |
| 0.83-0.93 | 0.88 | 1.00 | 2 | 0 | *DRB1*10:02* |
| 0.83-0.93 | 0.88 | 1.00 | 1 | 0 | *DRB1*10:12* |
| 0.12-2.79 | 0.59 | 0.73 | 18 | 2 | *DRB1*11:01* |
| 0.82-0.93 | 0.88 | 1.00 | 7 | 0 | *DRB1*11:02* |
| 0.83-0.93 | 0.88 | 1.00 | 1 | 0 | *DRB1*11:03* |
| 0.83-0.94 | 0.88 | 1.00 | 1 | 0 | *DRB1*11:04* |
| 0.83-0.93 | 0.88 | 1.00 | 3 | 0 | *DRB1*11:06* |
| 0.48-138 | 8.20 | 0.21 | 1 | 1 | *DRB1*11:37* |
| 0.83-0.94 | 0.88 | 1.00 | 1 | 0 | *DRB1*11:48* |
| 0.83-0.93 | 0.88 | 1.00 | 2 | 0 | *DRB1*11:50* |
| 0.83-0.94 | 0.88 | 1.00 | 1 | 0 | *DRB1*12:01* |
| 0.16-3.75 | 0.78 | 1.00 | 8 | 2 | *DRB1*13:01* |
| 0.83-0.93 | 0.88 | 1.00 | 2 | 0 | *DRB1*13:02* |
| 0.26-27.52 | 2.68 | 0.38 | 3 | 1 | *DRB1*13:07* |
| 0.06-0.17 | 0.10 | 0.11 | 0 | 1 | *DRB1*13:40* |
| 0.83-0.94 | 0.88 | 1.00 | 1 | 0 | *DRB1*13:70* |
| 0.83-0.94 | 0.88 | 1.00 | 1 | 0 | *DRB1*14:01* |
| 0.83-0.93 | 0.88 | 1.00 | 4 | 0 | *DRB1*14:02* |
| 0.24-5.97 | 1.22 | 0.68 | 6 | 2 | *DRB1*15:01* |
| 0.83-0.93 | 0.88 | 1.00 | 5 | 0 | *DRB1*15:02* |
| 0.83-0.94 | 0.88 | 1.00 | 1 | 0 | *DRB1*15:03* |
| 0.06-0.17 | 0.10 | 0.11 | 0 | 1 | *DRB1*15:04* |
| 0.83-0.93 | 0.88 | 1.00 | 4 | 0 | *DRB1*15:07* |
| 0.83-0.93 | 0.88 | 1.00 | 2 | 0 | *DRB1*16:01* |

**Table S7 The association between *HLA-A* in patients with drug reactions with eosinophilia and systemic symptoms (DRESS), compared to tolerance controls**

| **CI95%** | **OR** | **P value** | **CONTROL** | **PATIENTS** | *HLA-A* |
| --- | --- | --- | --- | --- | --- |
| 0.91-0.99 | 0.95 | 0.58 | 21 | 0 | *A*01:01* |
| 0.93-0.99 | 0.96 | 1.00 | 1 | 0 | *A*01:02* |
| 0.18-7.01 | 1.13 | 1.00 | 46 | 2 | *A*02:01* |
| 0.92-130.32 | 11 | 0.13 | 1 | 1 | *A*02:02* |
| 0.93-0.99 | 0.96 | 1.00 | 1 | 0 | *A*02:05* |
| 0.14-12.75 | 1.35 | 0.58 | 11 | 1 | *A*03:01* |
| 0.92-0.99 | 0.96 | 1.00 | 8 | 0 | *A*03:02* |
| 0.92-0.99 | 0.96 | 1.00 | 10 | 0 | *A*11:01* |
| 0.93-0.99 | 0.96 | 1.00 | 1 | 0 | *A*11:02* |
| 0.72-29.72 | 4.62 | 0.13 | 15 | 3 | *A*23:01* |
| 0.26-24.80 | 2.56 | 0.39 | 6 | 1 | *A*24:01* |
| 0.21-19.09 | 2 | 0.46 | 7 | 1 | *A*24:02* |
| 0.31-30.67 | 3.12 | 0.34 | 7 | 1 | *A*26:01* |
| 0.93-0.99 | 0.96 | 1.00 | 5 | 0 | *A*29:01* |
| 0.92-0.99 | 0.96 | 1.00 | 8 | **0** | *A*30:01* |
| 0.93-0.99 | 0.96 | 1.00 | 1 | **0** | *A*31:01* |
| 0.92-0.99 | 0.96 | 1.00 | 8 | **0** | *A*32:01* |
| 0.92-0.99 | 0.96 | 1.00 | 10 | **0** | *A*33:01* |
| 0.93-0.99 | 0.96 | 1.00 | 2 | **0** | *A*33:03* |
| 0.93-0.99 | 0.96 | 1.00 | 1 | **0** | *A*36:01* |
| 0.93-0.99 | 0.96 | 1.00 | 7 | **0** | *A*68:01* |
| 0.93-0.99 | 0.96 | 1.00 | 2 | **0** | *A*68:02* |
| 0.93-0.99 | 0.96 | 1.00 | 1 | **0** | *A*69:01* |

**Table S8 The association between *HLA-B* in patients with drug reactions with eosinophilia and systemic symptoms (DRESS), compared to tolerance controls**

| **CI95%** | **OR** | **P value** | CONTROL | PATIENTS | *HLA-B* |
| --- | --- | --- | --- | --- | --- |
| 0.93-0.99 | 0.96 | 1.00 | 6 | 0 | *B*07:01* |
| 0.39-39.77 | 3.96 | 0.28 | 10 | 1 | *B*07:02* |
| 0.93-0.99 | 0.96 | 1.00 | 2 | 0 | *B*07:05* |
| 0.44-46.49 | 4.57 | 0.25 | 5 | 1 | *B*08:01* |
| 0.93-0.99 | 0.96 | 1.00 | 4 | 0 | *B*08:02* |
| 0.93-0.99 | 0.96 | 1.00 | 3 | 0 | *B*08:27* |
| 0.93-0.99 | 0.96 | 1.00 | 4 | 0 | *B*13:01* |
| 0.93-0.99 | 0.96 | 1.00 | 2 | 0 | *B*13:02* |
| 0.93-0.99 | 0.96 | 1.00 | 6 | 0 | *B*14:02* |
| 0.93-0.99 | 0.96 | 1.00 | 2 | 0 | *B*15:01* |
| 0.92-0.99 | 0.96 | 1.00 | 1 | 0 | *B*15:02* |
| 0.93-0.99 | 0.96 | 1.00 | 5 | 0 | *B*15:03* |
| 0.93-0.99 | 0.96 | 1.00 | 1 | 0 | *B*18:01* |
| 0.93-0.99 | 0.96 | 1.00 | 3 | 0 | *B*18:02* |
| 0.93-0.99 | 0.96 | 1.00 | 4 | 0 | *B*27:01* |
| 0.92-0.99 | 0.95 | 1.00 | 12 | 0 | *B*35:01* |
| 0.92-0.99 | 0.96 | 1.00 | 8 | 0 | *B*35:02* |
| 0.93-0.99 | 0.96 | 1.00 | 4 | 0 | *B*35:03* |
| 0.51-55.75 | 5.37 | 0.22 | 2 | 1 | *B*38:01* |
| 0.93-0.99 | 0.96 | 1.00 | 2 | 0 | *B*38:02* |
| 0.93-0.99 | 0.96 | 1.00 | 1 | 0 | *B*39:01* |
| 0.93-0.99 | 0.96 | 1.00 | 2 | 0 | *B*39:02* |
| 0.35-34.67 | 3.50 | 0.31 | 4 | 1 | *B*40:01* |
| 3.50-245.32 | 29.33 | 0.01 | 1 | 4 | *B*40:02* |
| 0.93-0.99 | 0.96 | 1.00 | 4 | 0 | *B*40:04* |
| 0.93-0.99 | 0.96 | 1.00 | 3 | 0 | *B*40:06* |
| 0.92-0.99 | 0.96 | 1.00 | 10 | 0 | *B*41:01* |
| 0.93-0.99 | 0.96 | 1.00 | 3 | 0 | *B*41:02* |
| 0.93-0.99 | 0.96 | 1.00 | 1 | 0 | *B*42:01* |
| 0.93-0.99 | 0.96 | 1.00 | 1 | 0 | *B*42:02* |
| 0.93-0.99 | 0.96 | 1.00 | 3 | 0 | *B*44:01* |
| 0.93-0.99 | 0.96 | 1.00 | 8 | 0 | *B*44:02* |
| 0.93-0.99 | 0.96 | 1.00 | 3 | 0 | *B*44:03* |
| 0.92-0.99 | 0.96 | 1.00 | 11 | 0 | *B*49:01* |
| 0.93-0.99 | 0.96 | 1.00 | 2 | 0 | *B*49:02* |
| 0.44-46.49 | 4.57 | 0.25 | 6 | 1 | *B*50:01* |
| 0.44-46.49 | 4.57 | 0.25 | 5 | 1 | *B*50:02* |
| 0.93-0.99 | 0.96 | 1.00 | 5 | 0 | *B*50:12* |
| 0.92-0.99 | 0.96 | 1.00 | 8 | 0 | *B*51:01* |
| 0.93-0.99 | 0.96 | 1.00 | 1 | 0 | *B*51:04* |
| 0.93-0.99 | 0.96 | 1.00 | 1 | 0 | *B*51:51* |
| 0.93-0.99 | 0.96 | 1.00 | 3 | 0 | *B*52:01* |
| 0.93-0.99 | 0.96 | 1.00 | 2 | 0 | *B*52:02* |
| 0.93-0.99 | 0.96 | 1.00 | 1 | 0 | *B*53:01* |
| 0.93-0.99 | 0.96 | 1.00 | 3 | 0 | *B*55:01* |
| 0.93-0.99 | 0.96 | 1.00 | 2 | 0 | *B*58:01* |

**Table S9 The association between *HLA-DRB1* in patients with drug reactions with eosinophilia and systemic symptoms (DRESS), compared to tolerance controls**

| **CI95%** | **OR** | **P value** | **CONTROL** | **PATIENTS** | ***HLA-DRB1*** |
| --- | --- | --- | --- | --- | --- |
| 0.93-0.99 | 0.96 | 1.00 | 5 | 0 | *DRB1*01:01* |
| 0.93-0.99 | 0.96 | 1.00 | 2 | 0 | *DRB1*01:02* |
| 0.93-0.99 | 0.96 | 1.00 | 2 | 0 | *DRB1*01:03* |
| 0.08-7.15 | 0.77 | 1.00 | 20 | 1 | *DRB1*03:01* |
| 0.39-39.77 | 3.96 | 0.28 | 6 | 1 | *DRB1*03:02* |
| 1.23-223.46 | 16.62 | 0.10 | 3 | 1 | *DRB1*03:03* |
| 0.93-0.99 | 0.96 | 1.00 | 7 | 0 | *DRB1*04:01* |
| 0.93-0.99 | 0.96 | 1.00 | 5 | 0 | *DRB1*04:02* |
| 0.93-0.99 | 0.96 | 1.00 | 6 | 0 | *DRB1*04:03* |
| 0.93-0.99 | 0.96 | 1.00 | 2 | 0 | *DRB1*04:06* |
| 0.93-0.99 | 0.96 | 1.00 | 2 | 0 | *DRB1*04:14* |
| 0.93-0.99 | 0.96 | 1.00 | 1 | 0 | *DRB1*04:145* |
| 0.19-7.48 | 1.20 | 1.00 | 35 | 2 | *DRB1*07:01* |
| 0.93-0.99 | 0.96 | 1.00 | 2 | 0 | *DRB1*07:02* |
| 0.93-0.99 | 0.96 | 1.00 | 1 | 0 | *DRB1*08:01* |
| 0.93-0.99 | 0.96 | 1.00 | 3 | 0 | *DRB1*09:01* |
| 0.93-0.99 | 0.96 | 1.00 | 3 | 0 | *DRB1*10:01* |
| 0.93-0.99 | 0.96 | 1.00 | 2 | 0 | *DRB1*10:02* |
| 0.93-0.99 | 0.96 | 1.00 | 1 | 0 | *DRB1*10:12* |
| 0.91-0.99 | 0.95 | 0.58 | 18 | 0 | *DRB1*11:01* |
| 0.93-0.99 | 0.96 | 1.00 | 7 | 0 | *DRB1*11:02* |
| 1.76-636.74 | 33.50 | 0.07 | 1 | 1 | *DRB1*11:03* |
| 0.93-0.99 | 0.96 | 1.00 | 1 | 0 | *DRB1*11:04* |
| 0.93-0.99 | 0.96 | 1.00 | 3 | 0 | *DRB1*11:06* |
| 0.93-0.99 | 0.96 | 1.00 | 1 | 0 | *DRB1*11:37* |
| 0.93-0.99 | 0.96 | 1.00 | 1 | 0 | *DRB1*11:48* |
| 0.93-0.99 | 0.96 | 1.00 | 2 | 0 | *DRB1*11:50* |
| 0.93-0.99 | 0.96 | 1.00 | 1 | 0 | *DRB1*12:01* |
| 0.63-25.98 | 4.07 | 0.16 | 8 | 2 | *DRB1*13:01* |
| 0.92-130.32 | 11 | 0.13 | 2 | 1 | *DRB1*13:02* |
| 0.93-0.99 | 0.96 | 1.00 | 3 | 0 | *DRB1*13:07* |
| 0.93-0.99 | 0.96 | 1.00 | 1 | 0 | *DRB1*13:70* |
| 0.93-0.99 | 0.96 | 1.00 | 1 | 0 | *DRB1*14:01* |
| 0.93-0.99 | 0.96 | 1.00 | 4 | 0 | *DRB1*14:02* |
| 0.22-20.70 | 2.16 | 0.43 | 6 | 1 | *DRB1*15:01* |
| 0.93-0.99 | 0.96 | 1.00 | 5 | 0 | *DRB1*15:02* |
| 0.93-0.99 | 0.96 | 1.00 | 1 | 0 | *DRB1*15:03* |
| 0.93-0.99 | 0.96 | 1.00 | 4 | 0 | *DRB1*15:07* |
| 0.93-0.99 | 0.96 | 1.00 | 2 | 1 | *DRB1*16:01* |
